# Large-field irradiation techniques in Germany: A DGMP Working Group survey on the current clinical implementation of total body irradiation, total skin irradiation and craniospinal irradiation

**DOI:** 10.1016/j.zemedi.2024.09.002

**Published:** 2024-10-16

**Authors:** Lena Heuchel, Stephan Garbe, Armin Lühr, Maya Shariff

**Affiliations:** a Department of Physics, TU Dortmund University, Dortmund, Germany; b Department of Radiation Oncology, Universitätsklinikum Erlangen, Friedrich-Alexander-Universität Erlangen-Nürnberg (FAU), Erlangen, Germany; c Comprehensive Cancer Center Erlangen-EMN (CCC ER-EMN), Erlangen, Germany; d Department of Radiation Oncology, University Hospital Bonn, Bonn, Germany; e Department of diagnostic and interventional Radiology, University Hospital Bonn, Bonn, Germany

**Keywords:** Total body irradiation, Total skin irradiation, Craniospinal irradiation, Germany-wide survey

## Abstract

In 2023, a Germany-wide survey on the current clinical practice of three different large field irradiation techniques (LFIT), namely total body irradiation (TBI), total skin irradiation (TSI) and craniospinal irradiation (CSI), was conducted covering different aspects of the irradiation process, e.g., the irradiation unit and technique, dosimetrical aspects and treatment planning as well as quality assurance. The responses provided a deep insight into the applied approaches showing a high heterogeneity between participating centers for all three large field irradiation techniques. The highest heterogeneity was found for TBI. Here, differences between centers were found in almost every aspect of the irradiation process, e.g., the irradiation technique, the prescription dose, the spared organs at risk and the applied treatment planning method. For TBI, the only agreement was found in the fractionation scheme (2 Gy/fraction, 2 fractions/day) and the dose reduction to the lung. TSI was the rarest of the three LFITs. For TSI, the only agreement was found in the use of 6 MeV when irradiating with electrons. The reported approaches of CSI were closest to standard radiotherapy, using no CSI-specific irradiation techniques or treatment planning methods. For CSI, the only agreement was found in the prescribed dose to the brain (50 – 60 Gy). When asking for future requirements, participating centers considered the lack of standardization as the most important future challenge and suggested to perform (retrospective) patient studies. The results of such studies can then serve as a basis for new and improved guidelines.

## Introduction

Different large field irradiation techniques (LFIT) have been used for many years to irradiate large target volumes. The most common examples are total body irradiation (TBI), total skin irradiation (TSI), and craniospinal irradiation (CSI). There are many different approaches to perform TBI [1], [2], [3], [4], [5], [6], [7], [8], [9], [10], TSI [11], [12], [13], [14], [15], [16], [17], [18] or CSI [19], [20], [21], [22], [23], [24], [25], [26], [27], and several worldwide surveys have shown a high level of heterogeneity in the application of TBI [28], [29], [30], [31], [32], [33], [34], [35], TSI [28] and CSI [36]. Differences between centers include the definition of the target volume, dose limits for organs at risk (OAR), the prescription dose and fractionation scheme. As a result, there are no standardized or generally accepted planning and treatment approaches. With the increasing use of advanced irradiation techniques such as VMAT [23], [37], [38], [39], [40], [41], TomoTherapy [8], [9], [10], [13], [19], [21], [42], [43], [3], and proton irradiation [20], [24], [25], [26], [27] for large field irradiations, which allow more targeted irradiation and precise dose calculation, the need for standardization increases. Existing guidelines are highly descriptive and do not reflect the increasing use of modern irradiation techniques. Detailed knowledge of the current clinical practice is the first step in formulating new and improved guidelines. Therefore, the current study presents the results of a Germany-wide survey on the clinical approaches for TBI, TSI and CSI that are actually applied.

## Material and methods

In 2023, a questionnaire on the current clinical practice of LFIT was developed by the Working Group “Large Field Irradiation Techniques” of the German Society for Medical Physics (DGMP). The questionnaire (cf. Supplement) was designed as an online survey using LimeSurvey (LimeSurvey GmbH) and distributed via the DGMP mailing list. Centers responded between August and October 2023. The first questions related to personal information and which of the three LFITs the center performed. The main part of the survey asked for detailed information about each of the technique performed. Therefore, the main part was divided into three sections covering TBI, TSI, and CSI. Each of these sections was then subdivided into different topics covering general information, the irradiation equipment used, the irradiation technique, treatment planning, quality assurance and future requirements (Table 1). Some questions allowed the selection of more than one answer, and individual questions could be skipped. In addition, participants had the opportunity to comment on most questions.

**Table 1:** Number of questions (closed, open) of the main part of the survey for each section of the three techniques, namely total body irradiation (TBI), total skin irradiation (TSI), and cranio-spinal irradiation (CSI).

**Topic**	**Type**	**TBI**	**TSI**	**CSI**
General Information	Closed	2	2	1
	Open	2	2	3
	Total	4	4	4
Irradiation unit	Closed	3	1	1
	Open	4	2	1
	Total	7	3	2
Irradiation Technique	Closed	5	5	3
	Open	6	6	3
	Total	11	11	8
Treatment Planning	Closed	5	4	3
	Open	9	8	9
	Total	14	12	12
Quality Assurance	Closed	5	4	3
	Open	4	4	5
	Total	9	8	8
Future Requirements	Closed	-	-	-
	Open	5	5	5
	Total	5	5	5

Different strategies were used to analyze the responses, depending on the type of question. Closed questions (pre-defined answers) were analyzed quantitatively, while open-ended questions (free-text answers) were analyzed qualitatively, trying to find similarities and differences while considering the context of the answers given. In addition, similar responds from different centers were grouped and analyzed in a more quantitative manner.

## Results

**Figure 1:**
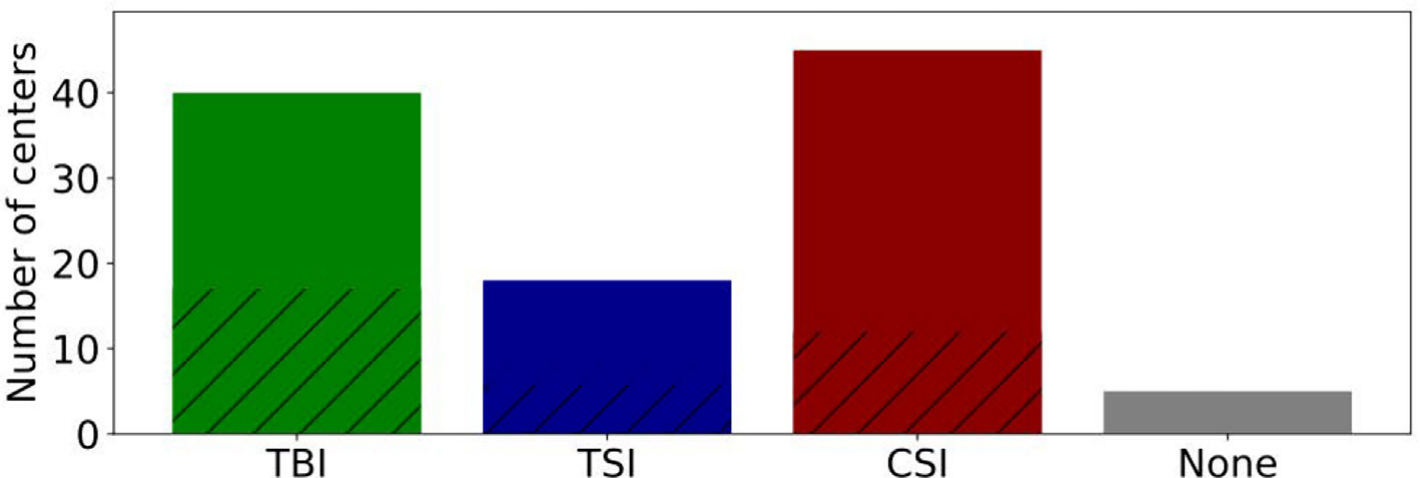
Number of centers performing total body irradiation (TBI), total skin irradiation (TSI), cranio-spinal irradiation (CSI), and none of the above-mentioned large field irradiation techniques. The hatched area indicates the number of centers providing details about their used irradiation technique.

75 centers answered at least one question of the questionnaire. The survey was mainly answered by a medical physicist (72 centers). However, 27 centers only responded to the question of which LFIT they perform and did not give any details on their clinical approaches. 40 centers reported to perform TBI, 18 TSI, and 45 CSI (Figure 1). Five centers reported that they did not perform any of the LFITs mentioned. Questions from the main part of the questionnaire, which asked for detailed information about the LFIT, were answered on average (± standard deviation) by 17 (± 3) centers for TBI, 6 (± 3) centers for TSI, and 12 (± 4) centers for CSI.

### Total body irradiation (TBI)

**Figure 2:**
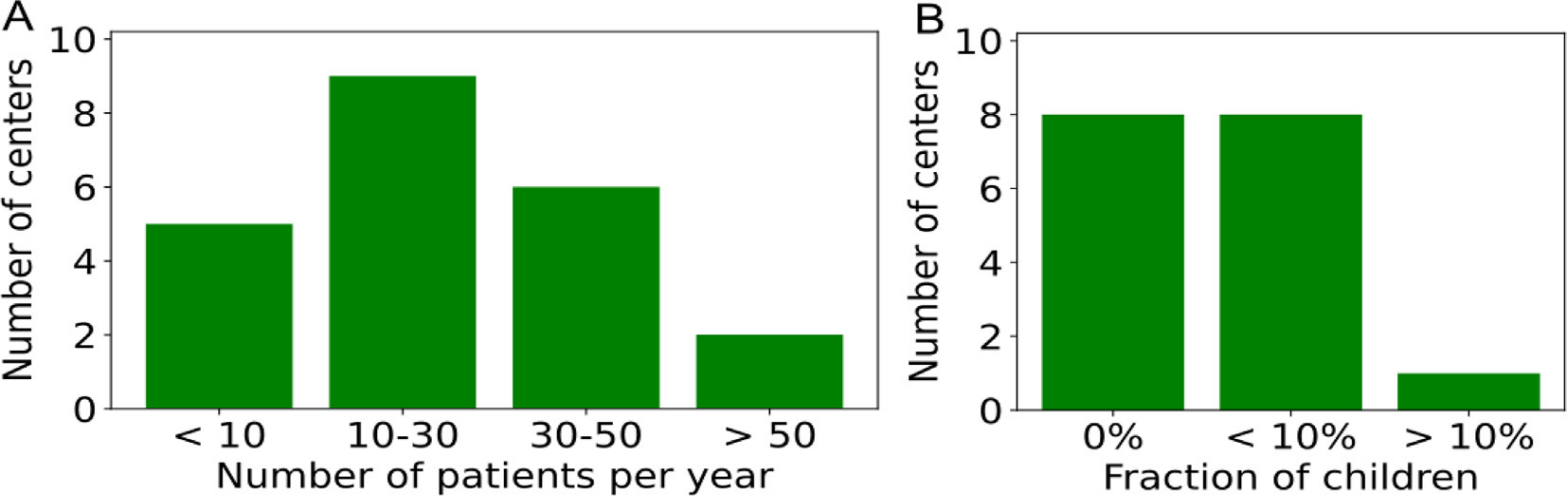
Number of patients per year (A) and proportion of children (B) for centers performing total body irradiation.

The most common response (9 centers) to the question of how many TBI patients a center treats per year was between 10 to 30 patients (Figure 2A). Although more than 90% of TBI patients at almost all centers were adults (Figure 2B), treatment of children was generally not excluded. TBI was most commonly used in leukemia patients (22 centers), followed by lymphoma patients (11 centers).

There were few differences between the participating centers in terms of the irradiation unit used and the energy delivered. 19 centers used a standard clinical linear accelerator (linac) for irradiation, while three centers used a TomoTherapy irradiation unit. 15 centers used 6 MV photons, and three centers varied the applied energy spectrum depending on the individual patient, sometimes using up to 18 MV to generate higher energies.

**Figure 3:**
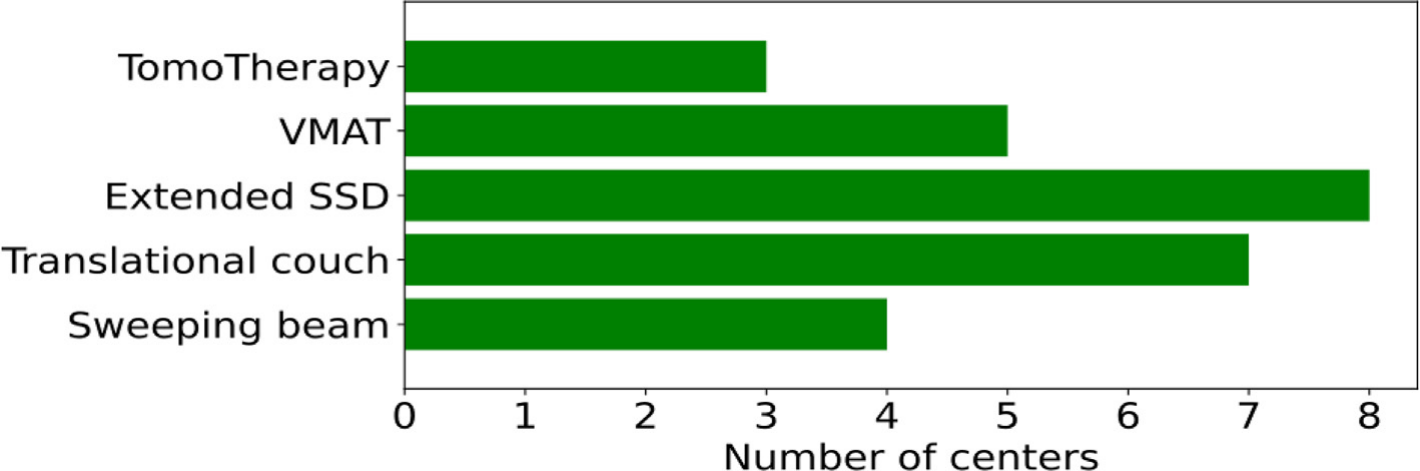
Number of centers using TomoTherapy, volumetric modulated arc therapy (VMAT), extended source-to-surface distance (SSD), the translational couch technique or a sweeping beam for total body irradiation.

A high degree of heterogeneity was found with respect to the technique used for total body irradiation (Figure 3). In eight centers, the technique used for TBI was irradiation with an extended SSD of up to 400 cm to create an effective field size large enough to cover the entire body. Seven centers used the translating couch technique with the patient lying on a couch close to the floor und being translated through the field. These centers performed treatment planning using the beam zone method [7]. The remaining techniques VMAT, sweeping beam and TomoTherapy were reported by 5, 4 and 3 centers, respectively. Chest wall boost with electrons was performed by three centers, while 15 centers reported that they never boosted the chest wall, four of them being centers using the VMAT or TomoTherapy techniques, which do not require chest wall boost.

When asked about the dose rate used at the level of the individual patient’s position, the answers varied widely between centers. Dose rates ranged from 5 to 125 cGy/min, with the highest dose rates typically used for VMAT, IMRT, or TomoTherapy. In addition, the dose rate was not constant for these techniques, whereas for the translating couch (as described in [7]) and extended source-to-surface distance (SSD) techniques, the same dose rate was usually applied to the entire body for all patients.

The question regarding patient positioning was answered by 15 centers. Most of the responders (11 centers) performed their irradiation in an anterior-posterior/posterior-anterior (appa) field orientation, using the prone and supine positions. Two centers irradiated their patients in a sitting position. The patient’s position was mostly ensured by the laser system in the irradiation room (7 centers). Five centers used radiochromic films, and three centers used a photostimulable phosphor (PSP) plate, specifically to ensure the correct position of the lung blocks. A complete treatment session, including positioning of the patient and equipment, took 30 min to one hour, while the irradiation itself lasted mostly less than 15 min (7 centers) and never exceeded one hour.

Twelve centers provided a detailed description of their irradiation techniques. Eight of them used a technique described in the literature, of which five described the translating couch technique [7] and one described a sweeping beam technique [44], [45], [46]. Two centers used a rotating tabletop on a standard linac couch for irradiation. These centers performed VMAT irradiations with 6 to 7 isocenters using overlapping 360° arcs with changing direction of rotation [41].

The other four centers that provided a detailed description performed a center-specific technique that did not follow any specific TBI method published in the literature. One center reported using the extended SSD technique with an SSD of approximately 400 cm, a bilateral field orientation, and a patient couch at the wall of the treatment room for lower prescription doses. For higher prescription doses, one fraction was applied with an appa irradiation using a patient couch on the floor, while all other fractions were applied with the same extended SSD technique as for lower prescription doses. For small children, this center used the standard treatment couch of the linac with an SSD of 120 cm, irradiating several appa fields. In another center, the patient lies on a carbon plate on the floor parallel to the direction of movement of the gantry. For irradiation, a generic rotation of the gantry was performed with a 40 cm × 40 cm field in the isocenter. Another way to perform TBI, as described by different center, was to irradiate in the prone and supine positions using three different gantry angles, one for the legs, one for the upper body, and one for the head with a field junction at the upper thighs and the shoulders. The remaining center that provided a detailed description of their technique used an arc technique, irradiating the patient in the prone and supine positions, with gantry angles ranging from 300° to 70°.

Regarding the treatment planning process, an overall high level of heterogeneity was found in almost all aspects. The prescribed dose ranged from 2 Gy to 12 Gy, and all 16 centers that responded to this question reported using at least two different prescription doses depending on the patient. The prescription dose was defined as the dose to a specific point (e.g., midpoint abdomen) by six centers and as the average dose to the target volume by seven centers, all of the latter calculated 3D dose distributions on a CT. Three centers used a mean dose value averaged over multiple points along the patient. All centers performing VMAT or TomoTherapy calculated 3D dose distributions on the patient CT, while most of the other centers (7 centers) used a patient CT to obtain quantities needed to determine the dose, such as patient thickness at a specified point, without performing a 3D dose calculation. Four centers reported that they never used a CT for treatment planning. Treatment planning time ranged from 10 min for extended SSD techniques to up to one day for TomoTherapy.

The only areas of agreement regarding treatment planning were the fractionation scheme and a dose reduction to the lung. All centers giving details on their fractionation scheme (14 centers) used 2 Gy per fraction and two fractions per day. Almost all centers (16 centers) reduced the dose to the lung, most of them to 8 Gy (5 centers) or 7 Gy (4 centers), using the lower value in the case of higher prescribed doses. Some centers also reduced the dose to the kidneys (3 centers) and lenses (2 centers). Only one center reported no OAR sparing. To achieve the desired lung dose reduction, lung blocks were used by eight centers. The use of beam spoilers during irradiation to shift the dose maximum to the skin surface was described by twelve centers and not limited to specific irradiation techniques.

Regarding the problem of machine failure, most centers (12) had two identical irradiation machines, the second of which could be used if the first was not working properly. Thus, there was no need for replanning in case of machine problems. Three centers reported that they used a different irradiation technique to perform TBI in case of machine failure, and therefore replanning was necessary. Most centers followed DGMP guidelines [48] (9 centers) or AAPM Report 17 [47] (6 centers) when performing TBI.

When asked about the advantages of the different techniques, centers considered the more standard techniques, such as extended SSD irradiation or the translating couch technique, to be robust, low-risk, and easy to implement and perform. Disadvantages of these techniques were considered to be the strong dependence of dose homogeneity on patient thickness and the need for additional dose measurements, since the irradiation conditions differ significantly from standard radiotherapy due to the extended SSD and the use of lung blocks. These lung blocks reduce the dose not only to the lung but also to the surrounding tissues, thereby compromising dose homogeneity. Moreover, the lung blocks had to be manufactured individually for each patient. In contrast, more modern techniques such as VMAT and TomoTherapy do not require lung blocks. As a result, the dose to the lung can be reduced without reducing the dose to the surrounding tissues. In addition, a more homogeneous dose can be achieved regardless of patient thickness, and no additional measurements are required as patients can be treated with a standard SSD. However, the latter techniques were considered less robust and more time consuming. Moreover, centers were concerned about using the modern techniques because of the segmented delivery and the much higher and less constant dose rates used. Since the target cells are present in the circulating blood, segmented irradiation with high dose rates may result in reduced target cell dose coverage compared to techniques that simultaneously irradiate larger volumes of the body.

The most important future challenge for TBI was the lack of standardization. Centers wanted more guidelines, especially regarding the definition of target volume, prescription dose, and dose limits for OAR. To formulate guidelines in the future, centers suggested retrospective patient studies that combine and analyze clinical outcome data from different centers.

### Total skin irradiation (TSI)

**Figure 4:**
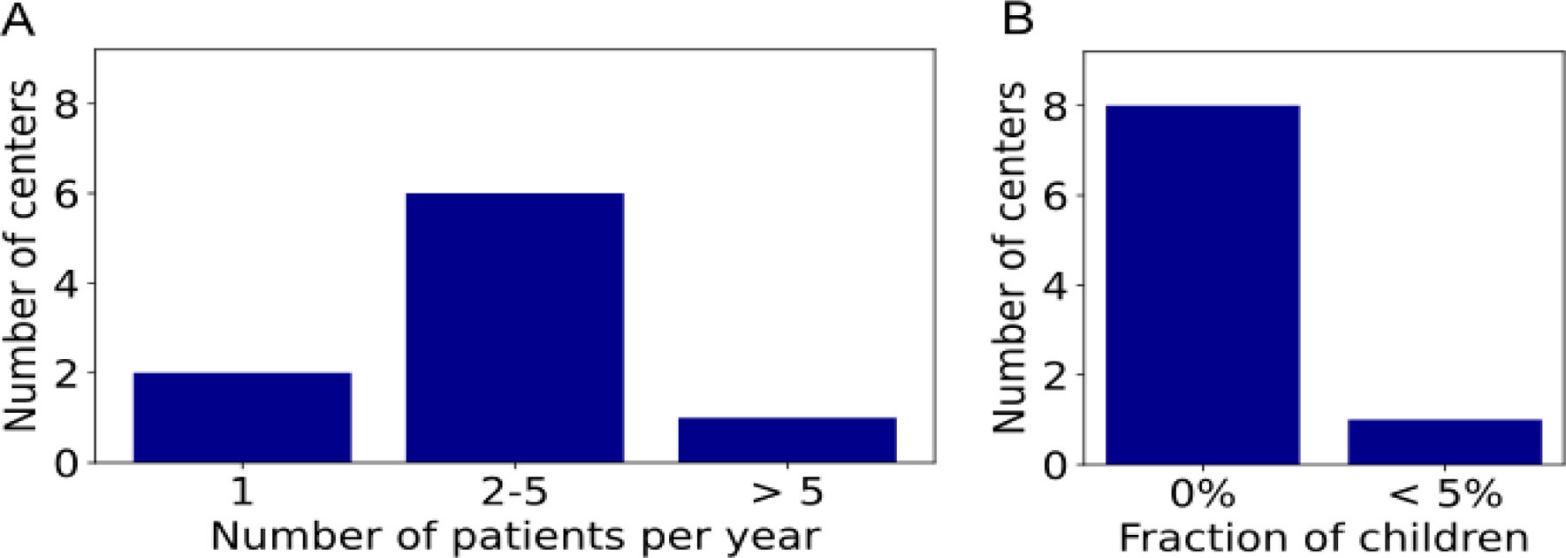
Number of patients per year (A) and proportion of children (B) for centers performing total skin irradiation.

Most centers (6) reported treating 2 – 5 patients per year with TSI (Figure 4A). Almost all centers (8) had never irradiated children (Figure 4B). TSI was usually indicated for patients with mycosis fungoides (10 centers) and cutaneous lymphoma (4 centers). Nine centers used their standard linac, most of them (6 centers) using 6 MeV electrons. Two centers used TomoTherapy to perform TSI using 2 MV photons.

**Figure 5:**
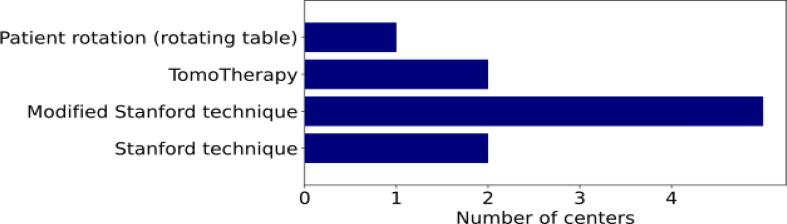
Number of centers using a rotating patient table, TomoTherapy, Modified Stanford Technique or the Standard Stanford Technique for total skin irradiation.

The most commonly used irradiation technique (5 centers) was the modified Stanford technique (Figure 5). Patients were usually irradiated in a standing position (8 centers). The palms and foot soles were irradiated by seven centers each, mostly using 6 MeV – 10 MeV electrons. Usually an SSD of 300 cm – 400 cm was used for TSI (7 centers). Most centers had special equipment for the patient to stand on and hold during treatment to make it as easy as possible for the patient to maintain the same position throughout the irradiation. Except for TomoTherapy, the patient position was ensured optically by using the laser system in treatment room. For TomoTherapy, vacuum cushions and masks were used to immobilize the patient. A complete treatment, including positioning, took 15 to 30 min at three centers and 30 to 45 min at three other centers. In two centers, the total treatment time was up to one hour. Irradiation itself took up to 10 min in four centers and up to 30 min in four other centers.

When asked about prescription doses and fractionation schemes, responses varied between centers. Centers used prescription doses between 12 and 30 Gy, using 1.5 Gy, 2 Gy, or 4 Gy per fraction. Typically (5 centers), one fraction per day was delivered, but two centers reported the delivery of two fractions per day or one fraction per day over two days. The target volume was defined as all or part of the skin, depending on the patient. The most commonly spared OARs were the eyes (5 centers), toes (4 centers), and fingernails (4 centers), usually with individual PMMA or lead absorbers. Furthermore, four centers reported the use of a beam spoiler during treatment. The time taken to prepare a treatment plan varied widely between centers. Two centers reported that it took one hour and two others that it took one day to develop a treatment plan. One center reported taking up to a week to complete a treatment plan.

Most centers (4) had an identical second irradiation machine to be used in case of machine failure. Two centers postponed treatment in the event of machine problems, as TSI is typically not time-critical. Except for TomoTherapy, for all other techniques, measurements during irradiation were mostly performed using ionization chambers or radiochromic films placed at various locations on the skin, including sites where dose deficits are most likely to occur. In the rotating table technique, the measurement tools were placed on a stand of the rotating unit in the isocenter plane during irradiation. Most centers (4) followed the AAPM report 23 [49] when performing TSI.

The Stanford technique was considered to be robust, less risky, and easy to use. But, at the same time, centers felt that this technique could lead to inhomogeneous dose distributions, requiring dose boosts, even in regions close to critical OARs. In addition, special equipment was required to perform TSI with this technique, and the patient had to be able to stand during the entire irradiation period. For the future, centers wanted to be able to apply a more homogeneous dose distribution to the skin either by further developing existing irradiation techniques or by developing new ones.

### Craniospinal irradiation (CSI)

**Figure 6:**
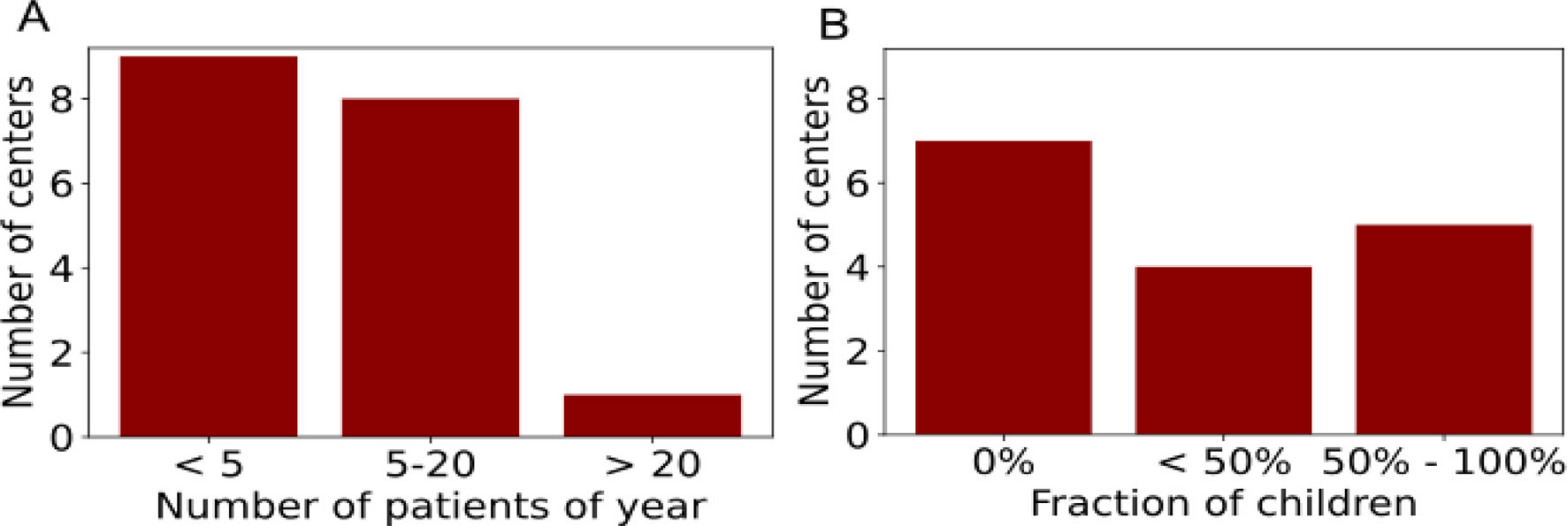
Number of patients per year (A) and proportion of children (B) for centers performing craniospinal irradiation.

Nine centers irradiated fewer than 5 patients per year with a CSI, while eight centers irradiated 5 to 20 patients per year (Figure 6A). Only one center treated more than 20 patients per year. There was a wide variation in the number of children treated with a CSI. Seven centers never irradiated children, while five centers irradiated up to 100% children (Figure 6B). CSI was indicated for many different diseases, the most common being medulloblastoma (3 centers), leptomeningeal spread (3 centers) and ependymoma (2 centers).

**Figure 7:**
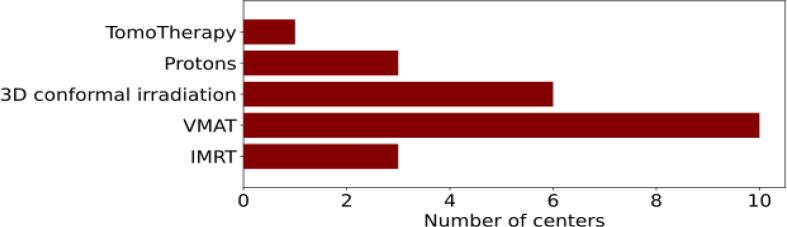
Number of centers using TomoTherapy, proton irradiation, 3D conformal irradiation, volumetric modulated arc therapy (VMAT), or intensity modulated radiation therapy (IMRT) for craniospinal irradiation.

Most centers used a standard linac to deliver CSI, with either the VMAT technique (10 centers), 3D conformal irradiation (6 centers), or IMRT (3 centers). Three centers used protons for irradiation, and one center used TomoTherapy (Figure 7). For patient positioning, most centers used cone beam CT (CBCT, 11 centers) and 3-degree-of-freedom couch (8 centers). Total treatment time, including patient positioning, was 30 min to one hour at seven centers and less than 30 min at four centers. The irradiation itself took less than 10 min in six centers and up to 40 min in the other six centers that answered this question.

The prescribed dose varied from 20 Gy to 60 Gy depending on the patient, but there was a consensus regarding the dose to the brain. In the case of lower prescription doses, almost all centers boosted the dose to the brain or parts of the brain to a total dose of 50 Gy to 60 Gy. The prescription dose was usually applied in 15 to 20 fractions using 1.5 to 2 Gy per fraction (8 centers), applying one fraction per day (12 centers). All centers performed target volume definition contouring, treatment planning and dose calculation always on a patient CT. The target volume was typically defined as the entire brain and spinal cord as well as a safety margin, which varied between 3 mm and 5 mm at different centers. Two centers also used MR images to aid in contouring and planning. Treatment planning time varied from 25 min to 1 h at one center, 2 to 5 h at six centers, and up to several days at three centers. All OAR close to the target volume were spared, with the lung and the optical system being most commonly cited. In general, the same dose limits as for standard radiotherapy were applied, the only exception being the spinal cord, where some centers used the prescription dose as the dose limit for this OAR. All centers reduced the dose in the OAR by using the corresponding objectives during plan optimization.

In case of machine failure, most centers (7) had an identical second irradiation unit to perform CSI. Five centers required a new treatment plan in case of machine failure. One of these prepared two treatment plans for each patient in advance. When asked about the advantages and disadvantages of the different techniques, the centers considered 3D conformal irradiation technique to be well known and established. However, patient positioning is critical for this technique to avoid under- and overdosing at the field boundaries. The VMAT technique, on the other hand, avoids field junction problems. However, the planning phase is more time consuming with the VMAT technique. The steep dose gradient has been cited as the greatest advantage of proton irradiation, but the treatment time may be longer with this technique than with others.

## Discussion

The survey showed a high degree of heterogeneity in the use of different LFIT in Germany. All three LFIT considered in this survey are rather rare treatments. The responding centers treated only about 20, 4, and 15 patients per year for TBI, TSI, and CSI, respectively. Although all three LFIT have been performed for many years, there are no commonly used planning and treatment approaches. Inter-center variability can be found in almost every aspect of the treatment process, including the dose prescribed, the definition of the target volume, the fractionation scheme, the spared OAR, the dose limits for the OAR, the irradiation technique used, the patient position, and the field orientation. For the three regarded LFITs, TBI showed the highest variation in the applied irradiation techniques resulting in an even higher heterogeneity compared to TSI and CSI.

Surveys on the clinical practice of TBI in other countries such as Belgium and the Netherlands [28], Canada [31], Australia and New Zealand [33], and Japan [29], [30] have also shown a high degree of heterogeneity. The number of centers performing TBI varied between 12 in Canada, 14 in Australia and New Zealand, 19 in the Netherlands and Belgium, and 186 in Japan. In our survey, 36 centers reported on performing TBI. Most of these centers treated approximately 10 to 30 patients per year, which is consistent with other countries showing that TBI is a rare treatment worldwide. In Japan, most centers used the extended SSD technique followed by the translating couch technique. In our survey, we found similar results for Germany. In other countries, almost all centers performed the extended SSD technique. Novel techniques such as VMAT are used much more frequently in Germany than any of the other countries. Therefore, in Germany, the number of centers defining the prescription dose as the dose to a specific point almost equaled to the number of centers defining the prescription dose as the average dose to the target volume, while in Belgium and the Netherlands, the prescription dose was usually defined as the dose to a specific point. Only two centers performed a 3D dose calculation on a patient’s CT. However, the surveys in the other countries were conducted between 2017 and 2021, while the German survey presented here was conducted in 2023. The number of centers adapting modern irradiation techniques might have also increased in the past years in the other regarded countries. In Japan, Australia and New Zealand, most centers performed bilateral irradiation with the patient in the supine position, whereas in Belgium and the Netherlands, most centers performed appa irradiation with the patient in the lateral decubitus position. In our study, most centers used appa field orientations in the prone and supine positions. Regarding the beam energy, most centers in Germany used 6 MV, which is consistent with the results from Australia and New Zealand. However, the most commonly used beam energy in Japan was 10 MV, while in Belgium and the Netherlands, 10 MV and 15 MV were the most common. The only agreement between the mentioned countries was found in the applied fractionation scheme (2 Gy per fraction and two fractions per day) and the routinely shielding of the lung during irradiation.

A survey on the clinical practice of TSI was conducted in Belgium and the Netherlands [28]. There, four centers reported to perform TSI compared to 17 centers in Germany. The number of patients per year ranged from 2 to 15, while in our survey the corresponding number ranged from 1 to 10. In Germany, an agreement regarding the used energy when irradiating with electrons was found. Almost all centers irradiated with electrons at 6 MeV, while in Belgium and the Netherlands the energy varied between 4 and 9 MeV. However, in Belgium and the Netherlands centers agreed on applying one fraction per day, using 1.5 Gy or 3 Gy per fraction. In Germany, a higher heterogeneity regarding the fractionation scheme was found. Centers used 1.5 Gy, 2 Gy, or 4 Gy per fraction, applying one fraction per day, two fractions per day, or two fractions over two days. In Belgium and the Netherlands, all centers used the Stanford technique, which was also the most commonly used technique in our survey. However, some centers in Germany had also sued other techniques such as TomoTherapy or irradiating the patient while standing on a rotating table leading to a higher heterogeneity in the applied TSI technique in Germany compared to Belgium and the Netherlands.

To our knowledge, there is no survey of clinical practice for CSI. A survey of patterns of care for medulloblastoma has been published [36] and found wide variation in the use of radiotherapy, particularly with regard to prescription dose and fractionation scheme. Our survey also found a high degree of variation in the prescription dose, ranging from 20 Gy to 60 Gy. The application of CSI was closer to standard radiotherapy protocols compared to TBI and TSI. For CSI, a 3D dose calculation was usually performed, using the same dose limits for the OAR as for standard radiotherapy, and the dose in the OAR was reduced during plan optimization by applying the corresponding objectives. Planning time varied significantly between centers, even for the same treatment technique. VMAT treatment planning took 25 min in one center, and up to several days in another. Novel techniques such as VMAT were more common for CSI than for TBI and TSI. CSI is the only LFIT for which the use of protons was reported.

While 69 centers responded to the question of which LFIT they perform, only a small fraction of these centers provided detailed information about their TBI, TSI, and CSI techniques. Many open-ended questions were used in the questionnaire, because we expected a wide variation in the used techniques. This allowed for detailed individual free-text answers, but also introduced the possibility of ambiguous answers. Moreover, in almost all centers, the questionnaire was answered by a medical physicist and thus represented only the ‘physical’ part of the treatment. To get a complete picture, physicians should be included in future studies and surveys.

The results of the survey highlight the high degree of variation in the use of TBI, TSI and CSI in Germany. Compared with other countries, this heterogeneity appeared to be even higher, as an increasing number of centers is implementing more advanced techniques such as VMAT, TomoTherapy and proton irradiation. The only agreements were found in the fractionation scheme and the shielding of the lung for TBI, the use of 6 MeV when irradiating with electrons for TSI and a dose of 50 to 60 Gy to the brain despite the prescription dose for the rest of the target volume for CSI, respectively. For all other aspects, no common agreement between centers was found, due to the highly descriptive character of existing guidelines and therefore the lack of standardization, which was considered to be the most important challenge for the future. To overcome this lack of standardization, centers suggested conducting retrospective patient studies that correlate various aspects of the treatment process, such as target volume definition, prescription dose, dose limits for OAR and 3D dose distributions, with relevant clinical endpoints, such as toxicities, survival, and the incidence of graft-versus-host disease. Because of the limited number of patient per center, combining clinical outcome data from different centers might be necessary to get statistically meaningful results The results of such studies could then serve as the basis for future guidelines for the use of different LFIT.

## Conclusion

The survey provided a detailed insight into the current clinical practice of different LFIT in Germany. It showed a high degree of heterogeneity in the application of TBI, TSI and CSI. Conventional techniques, such as extended SSD irradiation for TBI, Modified Stanford Technique for TSI and 3D conformal irradiation for CSI, were still most commonly used, but the number of centers using more advanced techniques (VMAT, TomoTherapy, and proton irradiation) seemed to be increasing. Therefore, the centers considered the lack of standardization as the most important future challenge. To overcome this, centers suggested that (retrospective) patient studies combining outcome data from different centers should be performed and the results should serve as a basis for new and improved guidelines.

## Declaration of interests

The authors declare that they have no known competing financial interests or personal relationships that could have appeared to influence the work reported in this paper.

## Conflicts of interest

The authors have no relevant conflicts of interest to disclose.
