# Supplementary material for: Large-field irradiation techniques in Germany: A DGMP Working Group survey on the current clinical implementation of total body irradiation, total skin irradiation and craniospinal irradiation
Source: Z Med Phys. 2024 Oct 16;36(1):16–25. doi: 10.1016/j.zemedi.2024.09.002 (PMC12901512; doi:10.1016/j.zemedi.2024.09.002)
Supplement: Supplementary Data 1 [file mmc1.pdf]

Umfrage Ganzkörperbestrahlung - AK Großfeldtechniken

Ziel der Umfrage ist eine Bestandsaufnahme der aktuell verwendeten Großfeldtechniken (Hier: Ganzkörperbestrahlung) in Deutschland. Die Umfrage richtet sich primär an physikalisch tätige Personen, wobei bei einzelnen Punkten auch medizinisches Personal zur Rate gezogen werden kann/sollte. Die Beantwortung der Fragen dauert ca. 15 Minuten und kann jeder Zeit unterbrochen und zu einem späteren Zeitpunkt fortgesetzt werden. Einzelne Fragen können übersprungen werden.

Vielen Dank, dass Sie sich die Zeit nehmen an dieser Umfrage teilzunehmen.

In dieser Umfrage sind 58 Fragen enthalten.

Allgemeine Fragen

1 Name des Zentrums

Bitte geben Sie Ihre Antwort hier ein:

[ ]

2 Berufliche Tätigkeit

❗ Bitte wählen Sie die zutreffenden Antworten aus:
Bitte wählen Sie alle zutreffenden Antworten aus:

- [ ] MPE
- [ ] MPE in Ausbildung
- [ ] Wissenschaftlicher Mitarbeiter/-in
- [ ] Doktorand/-in
- [ ] Student/-in
- [ ] Arzt/Ärztin
- [ ] Sonstiges: [ ]

3 E-Mail Adresse (für mögliche Rückfragen)

Bitte geben Sie Ihre Antwort hier ein:

4 Welche Großfeldtechniken werden in Ihrer Klinik durchgeführt?

❗ Bitte wählen Sie die zutreffenden Antworten aus:

Bitte wählen Sie alle zutreffenden Antworten aus:

- ☐ Ganzkörperbestrahlung
- ☐ Ganzhautbestrahlung
- ☐ Bestrahlung der Craniospinalen Achse
- ☐ Keine der genannten

Ganzkörperbestrahlung - Allgemeine Fragen

5 Wie viele Patienten pro Jahr werden in Ihrer Klinik mit einer Ganzkörperbestrahlung behandelt?

Bitte geben Sie Ihre Antwort hier ein:

6
Wie viel Prozent der mit einer Ganzkörperbestrahlung behandelten Pateinten in Ihrer Klinik sind Kinder?

Bitte geben Sie Ihre Antwort hier ein:

7 Bei welchen Erkrankungen wird eine Ganzkörperbestrahlung verschrieben?

❗ Bitte wählen Sie die zutreffenden Antworten aus:

Bitte wählen Sie alle zutreffenden Antworten aus:

- ☐ Leukämie
- ☐ Lymphome
- ☐ Multiple Myelome
- ☐ Plasmozytome
- ☐ Neuroblastome
- ☐ Sonstiges: ____________

8 Zu welchem Zeitpunkt wird die Ganzkörperbestrahlung durchgeführt?

❗ Bitte wählen Sie die zutreffenden Antworten aus:

Bitte wählen Sie alle zutreffenden Antworten aus:

- ☐ Vor der Chemotherapie
- ☐ Nach der Chemotherapie
- ☐ Zeitgleich mit der Chemotherapie
- ☐ Sonstiges: ____________

Ganzkörperbestrahlung - Bestrahlungsgerät

9 Mit welcher Bestrahlungsanlage führen Sie die Ganzkörperbestrahlung durch?

❗ Bitte wählen Sie die zutreffenden Antworten aus:

Bitte wählen Sie alle zutreffenden Antworten aus:

- ☐ Linac
- ☐ Cobalt-Gerät
- ☐ Tomotherapie
- ☐ Sonstiges: ______

10 Falls Sie die Bestrahlung an einem Linac durchführen, welchen Linac verwenden Sie?

❗ Kommentieren wenn eine Antwort gewählt wird

Bitte wählen Sie die zutreffenden Punkte aus und schreiben Sie einen Kommentar dazu:

- ☐ Varian ______
- ☐ Elekta ______
- ☐ Tomotherapie-Gerät ______
- ☐ Es wird kein Linac verwendet ______

Sonstiges: ______ ______

11 Welche Photonenenergie verwenden Sie für die Ganzkörperbestrahlung?

Bitte geben Sie Ihre Antwort hier ein:

12 Führen Sie eine Brustwandaufsättigung mit Elektronen durch?

❗ Bitte wählen Sie eine der folgenden Antworten:

Bitte wählen Sie nur eine der folgenden Antworten aus:

- ◯ Ja
- ◯ Nein
- ◯ Sonstiges

13 Falls Sie eine Brustwandaufsättigung mit Elektronen durchführen, welche Energie verwenden Sie?

Bitte geben Sie Ihre Antwort hier ein:

14 Welche Dosisrate des Bestrahlungsgeräts verwenden Sie für die Ganzkörperbestrahlung? (falls möglich bitte in cGy/min angeben)

Bitte geben Sie Ihre Antwort hier ein:

15 Welche Dosisrate liegt am Patienten vor? (falls möglich bitte in cGy/min angeben)

Bitte geben Sie Ihre Antwort hier ein:

Ganzkörperbestrahlung - Bestrahlungstechnik

16 Mit welcher Technik werden die Patienten bestrahlt?

❗ Bitte wählen Sie die zutreffenden Antworten aus:

Bitte wählen Sie alle zutreffenden Antworten aus:

- ☐ Sweeping Beam
- ☐ Translationsliege
- ☐ Stark vergrößerter Abstand zwischen Quelle und Patient
- ☐ VMAT/IMRT
- ☐ Tomotherapie
- ☐ Sonstiges:

17 Falls Sie die Bestrahlung mittels VMAT/IMRT durchführen, wie viele Felder und wie viele Patientenpositionen verwenden Sie?

Bitte geben Sie Ihre Antwort hier ein:

18 Welchen Abstand zwischen Quelle und Patient verwenden Sie?

Bitte geben Sie Ihre Antwort hier ein:

19 Welche Feldorientierung verwenden Sie?

❗ Bitte wählen Sie die zutreffenden Antworten aus:

Bitte wählen Sie alle zutreffenden Antworten aus:

- ☐ ap/pa
- ☐ RL/LR
- ☐ Beides (ap/pa und RL/LR)
- ☐ Keine der geannten
- ☐ Sonstiges:

20 In welcher Position befindet sich der Patient während der Bestrahlung?

❗ Bitte wählen Sie die zutreffenden Antworten aus:

Bitte wählen Sie alle zutreffenden Antworten aus:

- ☐ Bauchlage
- ☐ Rückenlage
- ☐ Seitenlage
- ☐ Sitzend
- ☐ Stehend
- ☐ Keine der genannten
- ☐ Sonstiges:

21 Wie erfolgt die Positionierung des Patienten?

Bitte geben Sie Ihre Antwort hier ein:

22 Wie erfolgt die Kontrolle der Positionierung?

❗ Bitte wählen Sie die zutreffenden Antworten aus:

Bitte wählen Sie alle zutreffenden Antworten aus:

- ☐ EPID
- ☐ Filme
- ☐ CBCT
- ☐ Sonstiges:

23 Wird der Patient auf der Standardliege des Beschleunigers bestrahlt?

❗ Bitte wählen Sie die zutreffenden Antworten aus:

Bitte wählen Sie alle zutreffenden Antworten aus:

- ☐ Ja
- ☐ Nein
- ☐ Sonstiges:

24 Wie lange ist die durchschnittliche Behandlungszeit (inkl. Positionierung, Bestrahlung)?

Bitte geben Sie Ihre Antwort hier ein:

25 Wie lange ist die reine Bestrahlungszeit?

Bitte geben Sie Ihre Antwort hier ein:

26 Bitte beschreiben Sie ihre Bestrahlungstechnik.

Bitte geben Sie Ihre Antwort hier ein:

Ganzkörperbestrahlung - Bestrahlungsplanung

27 Wie hoch ist die Verschreibungsdosis?

Bitte geben Sie Ihre Antwort hier ein:

28 In wie vielen Fraktionen wird die Verschreibungsdosis appliziert?

Bitte geben Sie Ihre Antwort hier ein:

29 Wie viele Fraktionen werden pro Tag appliziert?

Bitte geben Sie Ihre Antwort hier ein:

30 Wie wird die Verschreibungsdosis definiert?

❗ Bitte wählen Sie die zutreffenden Antworten aus:

Bitte wählen Sie alle zutreffenden Antworten aus:

- ☐ Mittlere Dosis im Zielvolumen
- ☐ Minimale Dosis im Zielvolumen
- ☐ Dosis an einem bestimmten Punkt (z.B. Mittelpunkt des Abdomen)
- ☐ Gemittelte Dosis über mehrere Punkte
- ☐ Sonstiges:

31 Wie wird das Zielvolumen definiert?

Bitte geben Sie Ihre Antwort hier ein:

32 Erfolgt die Bestrahlungsplanung auf Basis von einem CT?

❗ Bitte wählen Sie die zutreffenden Antworten aus:

Bitte wählen Sie alle zutreffenden Antworten aus:

☐ Es efolgt eine 3D Bestrahlungsplanung auf einem CT

☐ Es erfolgt eine "manuelle" Bestrahlungsplanung mithilfe von aus einem CT ermittelten Daten (z.B. Dicken)

☐ Nein

☐ Sonstiges: ____________

33 Falls die Bestrahlungsplanung auf Basis eines Ganzkörper-CTs erfolgt, beschreiben Sie bitte, wie das Ganzkörper-CT aufgenommen und erstellt wird.

Bitte geben Sie Ihre Antwort hier ein:

34 Welches Equipment wird während der Bestrahlung verwendet?

❗ Bitte wählen Sie die zutreffenden Antworten aus:

Bitte wählen Sie alle zutreffenden Antworten aus:

- [ ] Beamspoiler/Plexiglasplatte
- [ ] Abschirmblöcke
- [ ] Sonstiges: ____________

35 Wie lange dauert durchschnittlich die Erstellung eines Bestrahlungsplans?

Bitte geben Sie Ihre Antwort hier ein:

____

36 Wie lange dauern die Vorbereitungen vor der ersten Bestrahlung (inkl. Bestrahlungsplanung, Erstellen individueller Komponenten, ...)?

Bitte geben Sie Ihre Antwort hier ein:

____

37 Bitte beschreiben Sie, wie die Bestrahlungsplanung abläuft.

Bitte geben Sie Ihre Antwort hier ein:

Ganzkörperbestrahlung - Risikoorgane

38 Welche Risikoorgane/-strukturen werden geschont?

❗ Bitte wählen Sie die zutreffenden Antworten aus:

Bitte wählen Sie alle zutreffenden Antworten aus:

- ☐ Lunge
- ☐ Nieren
- ☐ Linsen
- ☐ Gar keine
- ☐ Sonstiges: ____________

39 Welche Dosisgrenzwerte werden für die einzelnen Risikoorgane verwendet?

Bitte geben Sie Ihre Antwort hier ein:

____

40 Wie werden die Risikoorgane geschont?

❗ Bitte wählen Sie die zutreffenden Antworten aus:

Bitte wählen Sie alle zutreffenden Antworten aus:

- ☐ Absorptionsblöcke / Transmissionsblöcke
- ☐ Verwendung des MLC
- ☐ Gar nicht
- ☐ Sonstiges: ____________

Ganzkörperbestrahlung - Qualitätssicherung

41 Welches Ausfallkonzept haben Sie?

❗ Bitte wählen Sie die zutreffenden Antworten aus:
Bitte wählen Sie alle zutreffenden Antworten aus:

- ☐ Identisches Bestrahlungsgerät in der Klinik
- ☐ Identisches Bestrahlungsgerät in anderer, nahegelegender Klinik
- ☐ Anderes Bestrahlungsgerät, an dem die gleiche Bestrahlungstechnik angewendet wird
- ☐ Komplett andere Bestrahlungstechnik
- ☐ Sonstiges: ____________

42 Ist bei Ausfall des Bestrahlungsgeräts eine Neuplanung nötig?

❗ Bitte wählen Sie die zutreffenden Antworten aus:
Bitte wählen Sie alle zutreffenden Antworten aus:

- ☐ Ja
- ☐ Nein
- ☐ Sonstiges: ____________

43 Welche Methoden/Messungen zusätzlich zur Standard-Qualitätssicherung führen Sie noch für die Ganzkörperbestrahlung durch?

Bitte geben Sie Ihre Antwort hier ein:

____

44 Führen Sie eine Dosismessung während der Bestrahlung durch?

❗ Bitte wählen Sie die zutreffenden Antworten aus:

Bitte wählen Sie alle zutreffenden Antworten aus:

- ☐ Ja
- ☐ Nein
- ☐ Sonstiges:

45 Welche Messgeräte werden für die Dosismessungen während der Bestrahlung verwendet?

Bitte geben Sie Ihre Antwort hier ein:

46 Wo werden die Messgeräte während der Bestrahlung platziert?

Bitte geben Sie Ihre Antwort hier ein:

47 Welche Risikoanalysen werden durchgeführt?

❗ Bitte wählen Sie die zutreffenden Antworten aus:

Bitte wählen Sie alle zutreffenden Antworten aus:

- ☐ FMEA
- ☐ Risikomatrix
- ☐ Gar nicht
- ☐ Sonstiges: ____________

48 Falls Sie Risikoanalysen durchführen, beschreiben Sie diese bitte.

Bitte geben Sie Ihre Antwort hier ein:

49 Folgen Sie bei Ihrer Bestrahlung bestimmten Richtlinien/Protokollen?

❗ Bitte wählen Sie die zutreffenden Antworten aus:

Bitte wählen Sie alle zutreffenden Antworten aus:

- [ ] Leitlinien der DGMP, 2003
- [ ] AAPM Report Nr. 17
- [ ] Richtlinien der ILROG, 2018
- [ ] Sonstiges: ____________

Ganzkörperbestrahlung - Allgemeine Einschätzungen

50 Welche Vorteile sehen Sie bei der von Ihnen verwendeten Technik im Vergleich zu anderen Techniken?

Bitte geben Sie Ihre Antwort hier ein:

51 Welche Nachteile sehen Sie bei der von Ihnen verwendeten Bestrahlungstechnik im Vergleich zu anderen Techniken?

Bitte geben Sie Ihre Antwort hier ein:

52 Was sehen Sie als die größte Herrausforderung bei der von Ihnen verwendeten Technik?

Bitte geben Sie Ihre Antwort hier ein:

53 Platz für zusätzliche Kommentare.

Bitte geben Sie Ihre Antwort hier ein:

Zukunft

54 Welche Forschungsfragen sollten Ihrer Meinung nach zukünftig zum Thema Ganzkörperbestrahlungen untersucht werden?

Bitte geben Sie Ihre Antwort hier ein:

55
Wären Sie prinzipielle interessiert daran, an einer in silico Patientenstudie teilzunehmen, bei der Sie einen Datensatz von einem Beispielpatienten erhalten würden und Sie einen Bestrahlungsplan für diesen Patienten erstellen müssten?

Bitte wählen Sie nur eine der folgenden Antworten aus:

- ◯ Ja
- ◯ Nein

Wir würden zukünftig, im Rahmen des AKs, gerne weiterführende Studien durchführen um die Unterschiede und Gemeinsamkeiten der einzelnen Techniken besser vergleichen zu können. Deswegen planen wir eine in silico Patientenstudie, bei der der Datensatz eines Beispielpatienten an verschiedene Zentren verteilt wird und diese eine Bestrahlungsplan nach Ihrer Technik erstellen. Auf dem nächsten AK Treffen werden wir hierauf noch detaillierter eingehen, dennoch würden wir gerne schon einmal abfragen, ob prinzipilles Interesse am Mitwirken an einer solchen Studie besteht.

56 Wären Sie prinzipiell interessiert daran, an einer dosimetrischen Studie teilzunehmen, bei der Sie die Dosis während der Bestrahlung an definierten Positionen entlang des Patienten messen müssten?

Bitte wählen Sie nur eine der folgenden Antworten aus:

- ◯ Ja
- ◯ Nein

Wir würden zukünftig, im Rahmen des AKs, gerne weiterführende Studien durchführen um die Unterschiede und Gemeinsamkeiten der einzelnen Techniken besser vergleichen zu können. Deswegen planen wir eine dosimetrische Studie, bei der die Dosis an definierten Punkten entlang des Patienten während der Bestrahlung gemessen werden soll. Auf dem nächsten AK Treffen werden wir hierauf noch detaillierter eingehen, dennoch würden wir gerne schon einmal abfragen, ob prinzipilles Interesse am Mitwirken an einer solchen Studie besteht.

57 Sind Sie damit einverstanden, dass ihr Zentrum inklusive der an Ihrem Zentrum durchgeführten Bestrahlungstechnik auf der Website des AKs gelistet wird?

Bitte wählen Sie nur eine der folgenden Antworten aus:

- ◯ Ja
- ◯ Nein
